# Adénome de la vésicule biliaire avec aspect cholestérolose

**DOI:** 10.11604/pamj.2016.24.147.9839

**Published:** 2016-06-18

**Authors:** Ben Slama Sana, Khadhar Aida

**Affiliations:** 1 Service d'Anatomie Pathologique, Hôpital M Slim, La Marsa, Tunisie; 2 Université de Tunis El Manar, Faculté de Médecine de Tunis, Tunis, Tunisie

**Keywords:** Gallbladder adenoma, dysplasia, cholecystitis, Gallbladder adenoma, dysplasia, cholecystitis

## Image en médecine

L'adénome de la vésicule biliaire est une tumeur bénigne souvent asymptomatique, découverte dans 0,5% des pièces de cholécystectomie. Nous présentons le cas d'une femme de 79 ans, admise pour suspicion de pancréatite aiguë d'origine lithiasique. Elle a bénéficié d'une cholécystectomie. A l'examen macroscopique, il existait une lésion jaunâtre au niveau du fond vésiculaire, de 1,7 cm de grand axe, végétante et friable. L'examen histologique montrait qu'une prolifération tumorale d'architecture papillaire et tubulaire, bordée par un épithélium pseudostratifié de type biliaire. La lamina propria était le siège de nombreux amas d'histiocytes spumeux. Le diagnostic était celui d'un adénome tubulopapillaire de type biliaire avec dysplasie de bas grade associé à des lésions de cholestérolose. Les adénomes de la vésicule biliaire affectent généralement les femmes adultes. Cliniquement, ils sont souvent asymptomatiques. Ils se présentent sous la forme de structures polyploïdes pouvant être sessiles ou pédiculées et se projetant dans la lumière de la vésicule biliaire. L’échographie ou l’échœndoscopie peuvent dans 5% mettre en évidence ces lésions et parfois en préciser la nature. Les polypes choléstéroliques toujours bénins, représentent environ la moitié des cas, ils sont généralement infracentimétriques. Dans notre cas, le diagnostic histologique était évident mais la présence d'histiocytes spumeux au niveau de la lamina propria était inhabituelle. La lésion entière doit être bien étudiée histologiquement afin de détecter des lésions de dysplasie de haut grade ou des signes d'invasion.

**Figure 1:**
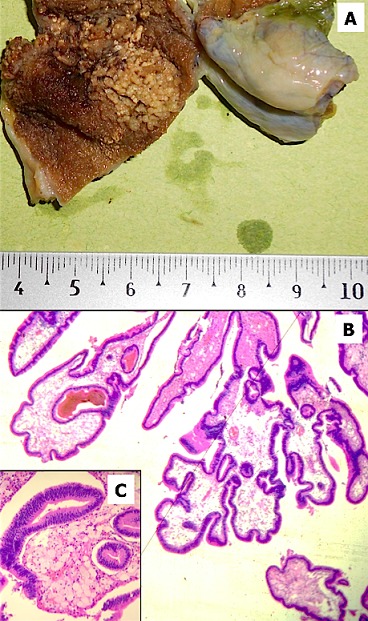
(A) aspect macroscopique: vésicule biliaire avec lésion jaunâtre végétante au niveau du fond, de 1,7 cm de grand axe; (B) aspect microscopique: prolifération tumorale d'architecture papillaire et tubulaire, bordée par un épithélium pseudostratifié de type biliaire; (C) aspect microscopique: présence de nombreux amas d'histiocytes spumeux dans la lamina

